# Identification and characterization of TrxA: a novel bacterial thioredoxin–metallothionein chimera

**DOI:** 10.1007/s00253-026-14002-w

**Published:** 2026-08-29

**Authors:** Annamaria Vitiello, Luciano Pirone, Martina Filocaso, Gabriella Fiorentino, Emilia Pedone, Danila Limauro

**Affiliations:** 1 Department of Biology, University of Naples Federico II, Via Cinthia, Naples, 89126 Italy; 2 Institute Biostructures and Bioimaging, C. N. R., Via Pietro Castellino 111, Naples, 80131 Italy; 3 Department of Environmental, Biological and Pharmaceutical Sciences and Technologies, University of Campania “Luigi Vanvitelli”, Caserta, 81100 Italy

**Keywords:** Metallothionein, Thioredoxin, Heavy metals, Bioremediation

## Abstract

**Abstract:**

Heavy metals (HMs) are naturally occurring elements which can be essential, such as zinc, copper, and iron, or non-essential, including cadmium, mercury, and lead. While essential metals serve as cofactors in critical enzymatic processes, elevated concentrations of both essential and non-essential HMs pose severe toxicity risks, primarily through oxidative stress, disruption of metal homeostasis, and biomolecular damage. Microorganisms have evolved diverse mechanisms to cope with metal-induced stress, including metal sequestration, enzymatic transformation, efflux systems, and surface immobilization. Among these, metallothioneins (Mts) are small, cysteine-rich proteins capable of high-affinity metal binding, contributing to cellular detoxification. Although Mts have been extensively studied in eukaryotes, knowledge of bacterial Mts remains limited, with characterized examples largely confined to cyanobacteria and a few other bacterial species. In this study, we identified a novel hybrid protein, TrxA, from *Runella aurantiaca*, containing a thioredoxin (Trx) domain fused to a Mt domain. The presence of the Trx domain may confer improved stability and solubility, supporting potential recombinant applications. In fact, the recombinant protein, named TrxMt, was heterologously expressed in *Escherichia coli*, displaying both disulfide-reducing activity and heavy metal–binding capability. Notably, TrxMt expression enhanced bacterial tolerance to multiple HMs, demonstrating its functional relevance in vivo. These findings expand the understanding of bacterial Mt diversity and suggest that TrxMt is a promising candidate for the bioremediation of heavy metal–contaminated environments, combining metal detoxification with favorable biochemical properties for industrial and environmental applications.

**Key points:**

• *Identification of TrxA, a novel hybrid thioredoxin–metallothionein in R. aurantiaca*

• *Recombinant protein TrxMt shows reductase activity and binds HMs*

• *Overexpression of TrxMt enhances tolerance to different HMs in E. coli*

**Supplementary information:**

The online version contains supplementary material available at https://doi.org/10.1007/s00253-026-14002-w.

## Introduction

Heavy metals (HMs) are commonly defined as metals or metalloids with relatively high density (> 5 g/cm^3^) and an atomic number > 20 (Chibuike and Obiora 2014). From a biological perspective, they can be distinguished as essential or non-essential trace elements. Essential HMs, such as zinc and copper, are important micronutrients for living organisms (Fraga 2005). However, at elevated concentrations, even essential metals can exert toxic effects. In contrast, non-essential HMs, such as cadmium, are considered highly toxic due to their deleterious effects on biological systems (Tchounwou et al. 2012).

HMs originate from both natural sources, such as geological and volcanic activity, and anthropogenic processes including mining and industrial practices. Due to their non-biodegradable nature, once released into the environment, HMs persist and progressively accumulate along the food web (Jaishankar et al. 2014), posing serious risks to ecosystems and human health (Hejna et al. 2018). At the cellular level, HM toxicity is primarily associated with the overproduction of reactive oxygen species, disruption of metal ion homeostasis, and damage to proteins and nucleic acids (Bruins et al. 2000).

The demand for technologies for heavy metal bioremediation is steadily increasing, driven both by the development of bioaccumulation systems for environmental remediation and by the identification of efficient adsorption methods that enable metal recovery (Diep et al. 2018). In this context, microbial bioremediation as an eco-friendly technological approach for the removal of HMs from contaminated environments is playing an increasingly significant role. Bacteria employ multiple mechanisms, including accumulation, adsorption, and transformation, to detoxify HMs in contaminated environments (Gallo et al. 2021). Numerous studies have highlighted the possibility of enhancing HMs accumulation in microorganisms through the expression of metal-binding proteins, by means of enzymes capable of producing metal-chelating peptides, through the engineering of bacteria designed to display high-affinity metal-binding proteins on their cell surface, enzymatic oxidoreductions and active export via efflux systems (Ahearn et al. 2004).

Mts belong to a superfamily of non-enzymatic proteins widely distributed among prokaryotes, lower eukaryotes, invertebrates, and mammals. They are very thermostable low-molecular-weight proteins (~ 3.5 to 14 kDa) enriched in cysteine residues arranged in recurring motifs such as C-X-C, C-X-X-C, and C–C, which bind metal cations with high affinity via thiol groups (Ziller and Fraissinet-Tachet 2018). Mt classification is primarily based on cysteine number and positional patterns; however, additional criteria, such as metal-binding preferences and overall amino acid composition, are also considered (Ziller et al. 2017). Although Mts were first identified in 1957 from the equine renal cortex (Margoshes and Vallee 1957) and have since been described in numerous eukaryotic organisms, knowledge of bacterial Mts remains limited (Robinson et al. 2001). Notably, bacterial Mts differ substantially from their eukaryotic counterparts in both the number and distribution of hydrophobic residues (Chatterjee et al. 2020). Mts have been mainly reported in cyanobacteria, including BMtA from *Oscillatoria brevis* (Liu et al. 2004) and SMtA from *Synechococcus* sp. (Robinson et al. 1990), which can bind zinc and cadmium ions. Mt proteins have also been identified in other bacteria, such as *Mycobacterium* spp. (Gold et al. 2008), *Salmonella* *enterica* (Naik et al. 2012) and *Pseudomonas* spp. (referred to as pseudothioneins), which have been shown to confer resistance to cadmium and lead (Habjanič et al. 2018).

This study aims to expand current knowledge of bacterial Mts. In this context, an interesting putative protein, TrxA, was found through in silico analysis in the genome of *Runella aurantiaca* (Yang et al. 2020), a Gram-negative bacterium isolated from the sludge of a manganese mine. The putative protein contains a thioredoxin (Trx) domain linked to an Mt domain, representing a naturally occurring chimeric protein. To the best of our knowledge, this type of fusion protein has not yet been biochemically characterized. Interestingly, the Trx domain may improve the thermostability and solubility of the protein, representing a potential advantage for recombinant protein production (Terpe 2003). Therefore, the focus of this study was to produce the *trxA* gene synthetically and investigate the biochemical and functional properties of the recombinant protein (TrxMt). The biochemical characterization of TrxMt may contribute not only to a better understanding of bacterial Mts but also to the identification of molecular features of interest for future biotechnological applications involving metal binding and sequestration.

## Materials and methods

### Bioinformatic analysis

Protein sequence alignment was performed using the BLASTp tool available at the National Center for Biotechnology Information (NCBI) (https://blast.ncbi.nlm.nih.gov/). Multiple sequence alignments (MSA) were generated with Clustal Omega (https://www.ebi.ac.uk/). Physicochemical parameters including molecular weight, theoretical isoelectric point (pI), extinction coefficient, and instability index, were predicted using the ProtParam tool from ExPASy (https://web.expasy.org/protparam/). The three-dimensional structure of TrxA was predicted using AlphaFold (https://alphafold.ebi.ac.uk/) (Fleming et al. 2025). Model confidence was assessed using the predicted Local Distance Difference Test (pLDDT) score provided by AlphaFold, which estimates the reliability of the predicted position of each amino acid residue.

### Gene synthesis

The trxA gene (WP_114464632) from *R. aurantiaca* (NCBI ID: 2282308) was synthesized by GenScript Biotech (Piscataway, NJ, USA). The nucleotide sequence was codon-optimized for expression in E. coli and cloned into the pET-28b(+) expression vector using *Nde*I and *Xho*I restriction sites (Supplementary Information 1). The resulting plasmid encoded a recombinant protein, designated TrxMt**,** bearing an N-terminal His_6_ tag.

### Expression and purification of TrxMt in *E. coli*

The recombinant vector pET28b-TrxMt was used to transform competent *E. coli* BL21(DE3) RIL cells. Transformants were selected on Luria–Bertani (LB) agar plates grown at 37 °C supplemented with kanamycin (50 µg/mL). A single colony was inoculated into LB medium and grown in an orbital shaker at 180 rpm until an OD_600_ of 0.5 was reached. Protein expression was induced by adding 0.5 mM isopropyl-β-d-thiogalactopyranoside (IPTG) for 18 h at 37 °C. Cells from 1 L culture were harvested by centrifugation at 3200 × g for 15 min at 4 °C and stored at− 20 °C overnight. The pellet was resuspended in 50 mL of lysis buffer containing 20 mM Tris-HCl pH 8.2, 10 mM NaCl, 0.2 mM phenylmethylsulfonyl fluoride (PMSF), 1 mM DTT and 2 mg/mL lysozyme. The mixture was incubated for 1 h at 37 °C with gentle shaking. DNase I was added to a final concentration of 5 µg/mL, and the mixture was incubated for an additional 45 min at 4 °C. Cells were lysed by three cycles of freeze–thaw: a rapid freezing in ethanol/dry ice for 20 min followed by incubation at 37 °C for 15 min. The lysate was clarified by centrifugation at 15,500 × g for 45 min at 4 °C, and the soluble fraction underwent thermal precipitation at 60 °C for 30 min, followed by centrifugation at 15,500 × *g* for 45 min at 4 °C. The resultant supernatant was stored at 4 °C overnight for subsequent purification steps. Anion-exchange chromatography was performed using a HiTrap Q XL 1 mL column (Cytiva, Global Life Sciences Solutions USA) connected to an ÄKTA pure system. The column was equilibrated with buffer A (20 mM Tris–HCl pH 8.3, 10 mM NaCl), and the elution was carried out with a linear gradient from 0 to 100% of buffer B (20 mM Tris–HCl pH 8.3, 1 M NaCl). Peak fractions were pooled and applied to a HisTrap HP 1 mL column (Cytiva). The column was equilibrated with buffer A (20 mM Tris-HCl pH 7.5, 300 mM NaCl, 10 mM imidazole), and the elution was performed using a step gradient of 10%, 50%, and 100% of buffer B (20 mM Tris–HCl pH 7.5, 300 mM NaCl, 500 mM imidazole). The peak fractions were pooled and dialyzed against Tris-HCl 20 mM pH 7.5 and NaCl 100 mM at 4 °C for 16 h. TrxMt purity degree was assessed by 15% SDS-PAGE followed by Coomassie Brilliant Blue R-250 staining. The protein concentration was determined using the Bradford assay with bovine serum albumin as a standard. Purified fractions were supplemented with 1 mM DTT.

### Light scattering measurements

The native molecular weight of the purified TrxMt was analyzed by Static Light Scattering (SLS) equipment consisting of a size-exclusion chromatography coupled to a Mini DAWN Treos spectrometer (Wyatt Instrument Technology Corp.) connected to a triple-angle light scattering detector, equipped with a Quasi-Elastic Light Scattering (QELS) module. One milligram of protein was loaded onto Superdex 75 10/300 column (Cytiva), equilibrated in 20 mM Tris-HCl pH 7.5 and 150 mM NaCl at a flow rate of 0.5 mL/min. Data were analyzed using Astra 5.3.4.14 software (Wyatt Technology, Santa Barbara, CA, USA).

Dynamic LS (DLS) measurements were performed using a Malvern Nano Zetasizer (Malvern, UK). Samples (100 µL) of TrxMt at 0.04 mM and 1.5 mM were loaded into disposable cuvettes and analyzed at 25 °C. Each measurement was recorded three times with 11 subruns using the multimodal mode. The *Z*-average diameter was calculated from the correlation function using the Malvern technology software ZS Xplorer version 3.2.0.84.

### Insulin disulfide reduction assay

The reductase activity of TrxMt was evaluated using a turbidimetric assay based on the reduction of insulin disulfide bonds, leading to precipitation of the insoluble insulin B chain. The reaction mixture contained 0.1 M phosphate buffer pH 7.0, 2 mM EDTA pH 8.0, 1 mg/mL insulin, and 8 µM TrxMt. A total of 1 mM dithiothreitol (DTT) was added immediately before initiating the reaction. A negative control containing all the components except TrxMt was included. Absorbance was monitored at 650 nm for 60 min at 30 °C using an Agilent Varian Cary 50 spectrophotometer.

#### Far-UV circular dichroism analysis of TrxMt in the presence of HMs

Circular dichroism (CD) spectra were measured using a Jasco J-815 spectropolarimeter at room temperature using a 0.1 cm path length cell. The far-UV CD spectra were recorded from 190 to 300 nm using 1 µM of apo-TrxMt in 5 mM Tris-HCl pH 7.5 and 1 mM TCEP. The concentrations of Cd^2^⁺, Cu^2^⁺, and Zn^2^⁺ were selected based on preliminary metal titration experiments performed over the range of 40–120 µM. Subsequently, HMs were added at the following concentrations: 60 µM CdCl_2_, 120 µM CuCl_2_, and 80 µM ZnCl_2_. Three acquisitions were recorded for each spectrum at a scan rate of 50 nm/min, 0.5 nm bandwidth. Baseline correction was performed by subtracting the buffer contribution. The CD data were smoothed using a Savitzky-Golay filter, avoiding distortion of spectral features, and converted in Delta epsilon (Δε).

expressed in nM^−1^ cm^−1^. It was calculated using the following equation:

$$\Delta\upvarepsilon =\frac{[\Theta ]}{32980\cdot c \cdot l}$$

where *[Θ]* is the raw ellipticity measured in millidegrees (mdeg), *c* is the protein concentration (mg/mL) and *l* is the optical path length of the cell (cm). Quantitative secondary structure estimation was carried out using the BeStSel website (Micsonai et al. 2021) (https://bestsel.elte.hu/sexamin.php). The quality of spectral fitting was assessed using the normalized root mean square deviation (NRMSD). Fits with NRMSD values < 0.1 were considered reliable.

### Metal-binding analyses by isothermal titration calorimetry

Isothermal Titration Calorimetry (ITC) studies were performed at 25 °C using a MicroCal PEAQ-ITC (Malven Panalytical, Malvern, UK). Titrations were conducted using 600 µM CdCl_2_, 500 µM CuCl_2_, or 1 mM ZnCl_2_ as ligands and 20 µM TrxMt in the cell. Both protein and metals were prepared in 20 mM Tris-HCl, pH 7.5. Injections of 1.5 µL (first injection 0.3 µL), for a total of 55 for CdCl_2_ and ZnCl_2_ and 100 for CuCl_2_, were performed at 120-s intervals with stirring at 1000 rpm. Control titrations were conducted by injecting metal solutions into the buffer. The data were processed using MicroCal PEAQ-ITC Analysis software and imposing a one-site binding model for titrations experiments with CdCl_2_, ZnCl_2_ and CuCl_2_. PEAQ-ITC Analysis software measures the association constant (*K*), stoichiometry (*n*) and the enthalpy change (Δ*H*) of binding. *K* is related to the Gibbs free energy of binding (Δ*G*), so the entropy change (Δ*S*) of the reaction can thus be derived from the relationship Δ*G* = −RTlnKD = Δ*H* − TΔS.

### Metal tolerance of *E. coli* cells expressing TrxMt

Preliminary minimum inhibitory concentration (MIC) assays were conducted to assess the sensitivity of *E. coli* BL21(DE3) RIL transformed strains to the tested metal ions, The cells were cultured in LB medium supplemented with kanamycin (50 µg/mL) and chloramphenicol (30 µg/mL) at 37 °C. Protein expression was induced with 0.5 mM IPTG at an OD_600_ of 0.2. After induction for 30 min, CdCl_2_, ZnCl_2_, or CuCl_2_, were added to final concentrations ranging 0.5 mM–5 mM for each HMs. Growth was monitored by measuring the optical density at 600 nm (OD_600_) after 15 h at 37° with continuous orbital shaking.

The tolerance of *E. coli* cells expressing TrxMt to HMs was evaluated by monitoring growth kinetics of BL21(DE3) RIL cells harboring either pET-28b(+)-TrxMt or pET-28b(+), at subinhibitory concentrations of 2 mM Zn^2^⁺, 0.8 mM Cd^2^⁺, and 3.5 mM Cu^+2^ for 15 h by measuring the optical density at 600 nm (OD_600_) using a Synergy HTX microplate reader (BioTek) at 37 °C with continuous orbital shaking at 200 rpm. Data are presented as mean ± SD of three independent biological replicates.

## Results

### In silico analysis of TrxA

**Fig. 1:**
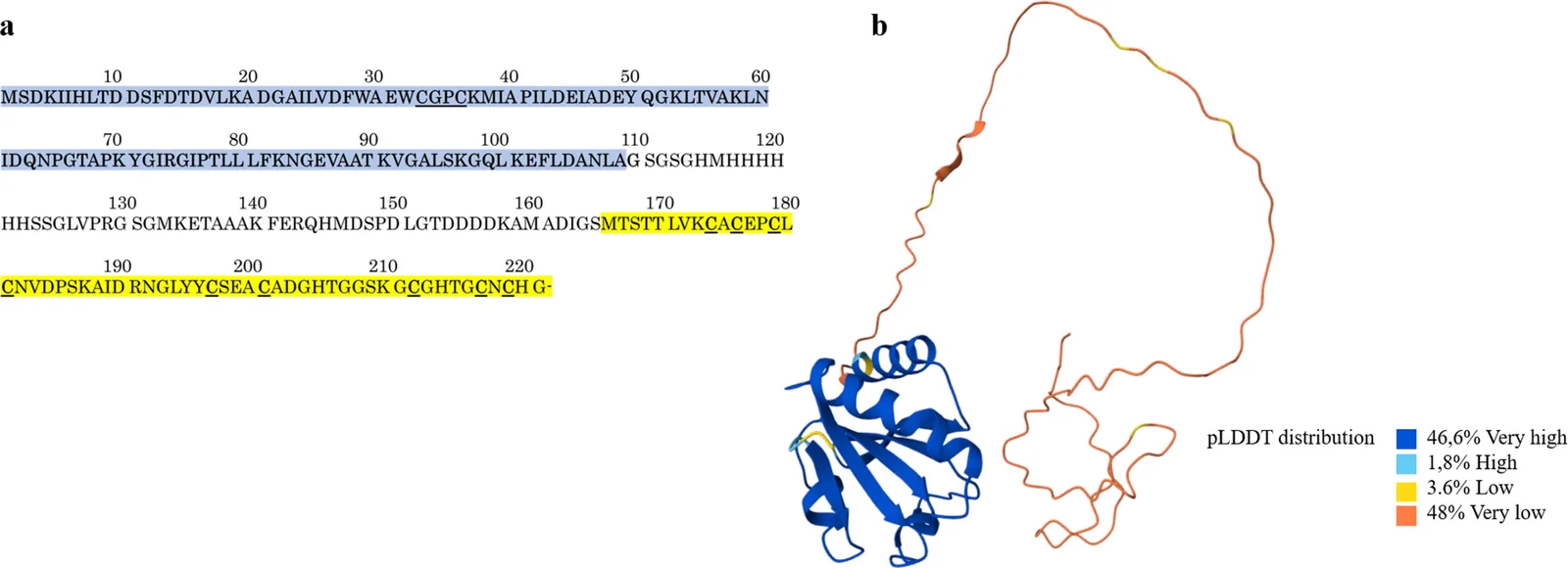
Primary sequence and AlphaFold-predicted structure of TrxA from *R. aurantiaca*. **a** The N-terminal region (residues, 1–109), shown in blue, corresponds to the putative Trx sequence and contains the canonical C-X-X-C motif underlined. The C-terminal region (residues, 165–221), shown in yellow, corresponds to a putative Mt sequence, with cysteine residues underlined. **b** AlphaFold-predicted three-dimensional structure of TrxA colored according to the pLDDT confidence score, ranging from very low (orange) to very high (blue) confidence

BLASTp and AlphaFold analyses were carried out to identify novel microbial Mts, revealing a previously uncharacterized protein from *R. aurantiaca* (Yang et al. 2020), TrxA (accession number RDB02259.1). Sequence analysis showed that TrxA is composed of two distinct regions: an N-terminal sequence homologous to thioredoxin (Trx) (residues, 1–109) and a C-terminal cysteine-rich region (residues, 165–221) typical of Mts (Fig. 1a). The AlphaFold-predicted three-dimensional structure of TrxA (Fig. 1b) revealed a canonical Trx fold at the N-terminus, consisting of a central four-stranded β-sheet flanked by three α-helices (Pedone et al. 2010), connected by an unstructured linker region to a C-terminal Mt domain. The AlphaFold model exhibited high confidence scores (pLDDT > 90) throughout most of the Trx domain, whereas lower confidence values were observed within the Mt domain. This reduced confidence likely reflects the intrinsic structural flexibility of Mts and their tendency to adopt a more defined conformation upon metal binding.

**Fig. 2:**
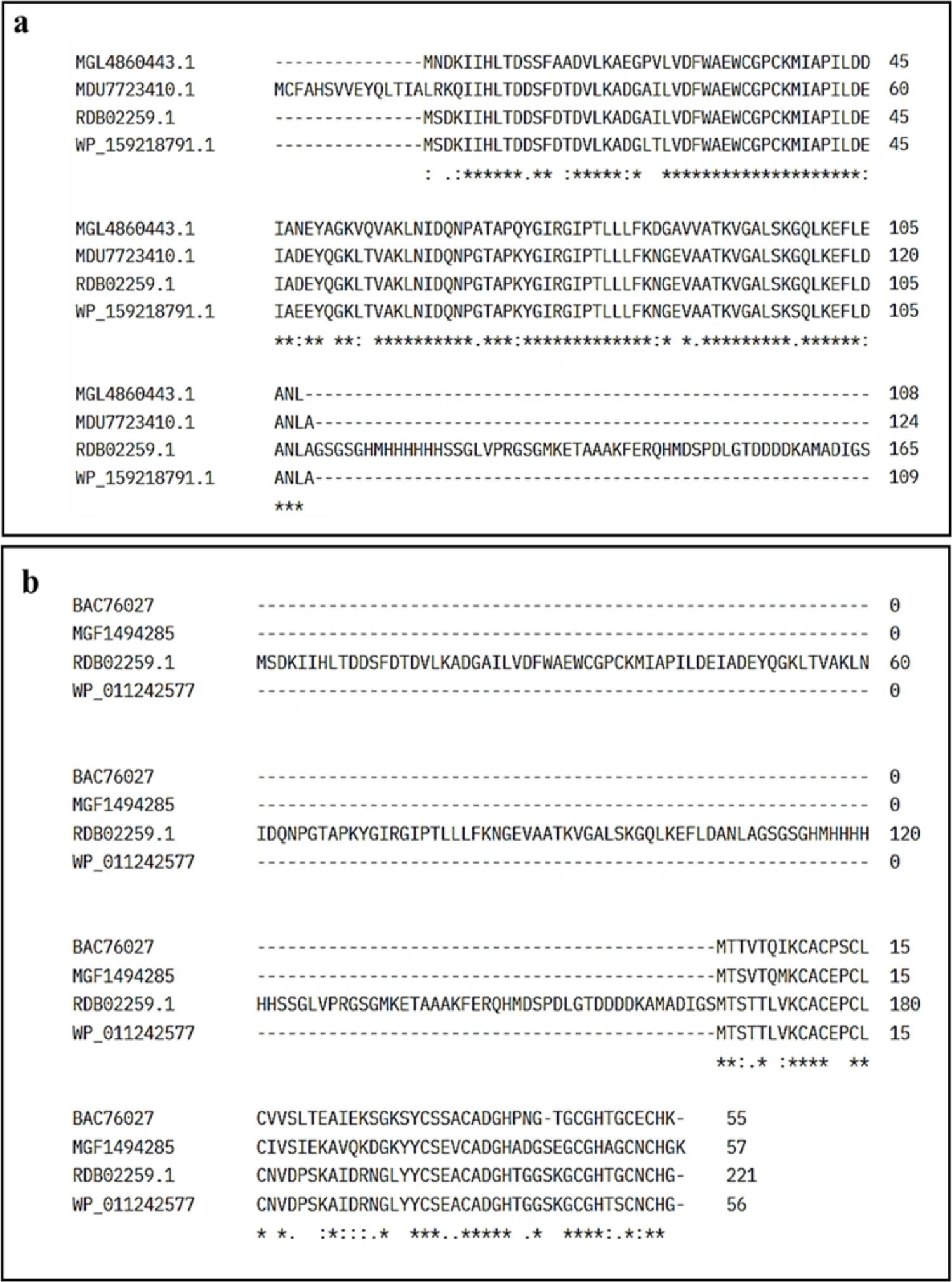
Clustal Omega multiple sequence alignment of TrxA (RDB02259.1). **a** MS alignment of N-terminal sequence of TrxA (RDB02259.1) with three Trxs from different species: TrxA from *K. pneumoniae* (WP_159218791.1); TrxA from Citrobacter sp (MDU7723410.1). TrxA from Enterobacteriaceae bacterium (MGL4860443.1). Asterisks (*) indicate fully conserved residues, while colons (:) and dots (.) indicate strong and weak conservation, respectively. **b** Clustal Omega multiple sequence alignment of C-terminal sequence of TrxA (RDB02259.1) with three Mts from different species: Mt from *S. elongatus* (WP_011242577), Mt from *M. cyanobacterium* (MGF1494285), and BMtA from *O. brevis* (BAC76027). Asterisks (*) indicate fully conserved residues, while colons (:) and dots (.) indicate strong and weak conservation, respectively

BLASTp analysis of the N-terminal region revealed high sequence similarity to bacterial Trxs, with 96% identity to TrxA from *Citrobacter* sp. (locus MDU7723410), 95% identity to TrxA from *Klebsiella pneumoniae* (locus WP_159218791.1), and 83% identity to TrxA from Enterobacteriaceae bacterium (locus MGL4860443.1). Multiple sequence alignment performed using Clustal Omega confirmed strong conservation within this region (Fig. 2a), including the canonical C-X-X-C catalytic motif, preserved across all aligned sequences (Carvalho et al. 2006), which is essential for Trx-mediated disulfide bond reduction (Limauro et al. 2009)..

The Trx-homologous region is separated by a 56-residue linker from the C-terminal sequence (residues, 166–221). This region is enriched in cysteine residues (15.7%), arranged in characteristic C-X-C, C-X-X-C, C-X-X-X-C, and C-X-X-X-X-C motifs, commonly associated with Mts, suggesting a potential role in metal coordination. BLASTp analysis of this C-terminal region revealed strong similarity to bacterial Mts, including Mt from *Synechococcus elongatus* with 98.21% identity (locus WP_011242577), Mt from a *Microcoleaceae cyanobacterium* with 69.64% identity (locus MGF1494285), and BMtA from *O. brevis* with 60% identity (locus BAC76027). Multiple sequence alignment performed using Clustal Omega highlighted a high degree of conservation across this cysteine-rich region (Fig. 2b).

In a comparative analysis, as shown in Table 1, the Mt sequence of TrxA shares many structural features with canonical bacterial Mts (Chatterjee et al. 2020), including length, cysteine content, amino acid composition as well as the prevalence of lysine over arginine, and similar serine/threonine and isoleucine/leucine ratios. The high sequence similarity of the C-terminal region of TrxA with other bacterial Mts identified through Blastp analysis, together with the conserved cysteine motifs and structural features, indicates its classification as a bacterial Mt.

**Table 1:** Canonical features of bacterial Mts compared to the Mt domain of TrxA

	Bacterial Mt	TrxA Mt domain
Amino acids	~60	56
Cys (%)	~16%	15.7%
Lys/Arg	Lys > Arg	Lys (3) > Arg (1)
Ser/Thr	Ser < Thr	Ser (4) < Thr (5)
Ile/Leu	Ile ≤ Leu	Ile (1) ≤ Leu (3)

Furthermore, ProtParam analysis predicted the physicochemical properties of TrxA, including an isoelectric point (pI) of 5.5, a molecular weight of 23,444 Da, and a low instability index (II) of 20.98, indicating that the TrxA is comparatively more stable than its Mt domain, which exhibits an II of 31.49.

### Expression, purification, and quaternary structure of recombinant TrxA (TrxMt)

The synthetic *trxA* gene was cloned into the pET-28b(+) expression vector, and the recombinant protein, designated TrxMt and bearing an N-terminal His_6_ tag, was heterologously expressed in *E. coli* BL21(DE3) RIL cells. Predicted physicochemical parameters of TrxMt (241 amino acids) were calculated using ProtParam. The protein has a molecular weight of 25,607 Da and a theoretical pI of 6.05. Extinction coefficients were calculated for both the reduced (16.960 M^−1^·cm^−1^) and oxidized (17.585 M^−1^·cm^−1^) forms of cysteine residues. The instability index (21.81) suggests that TrxMt is predicted to be stable in vitro.

**Fig. 3:**
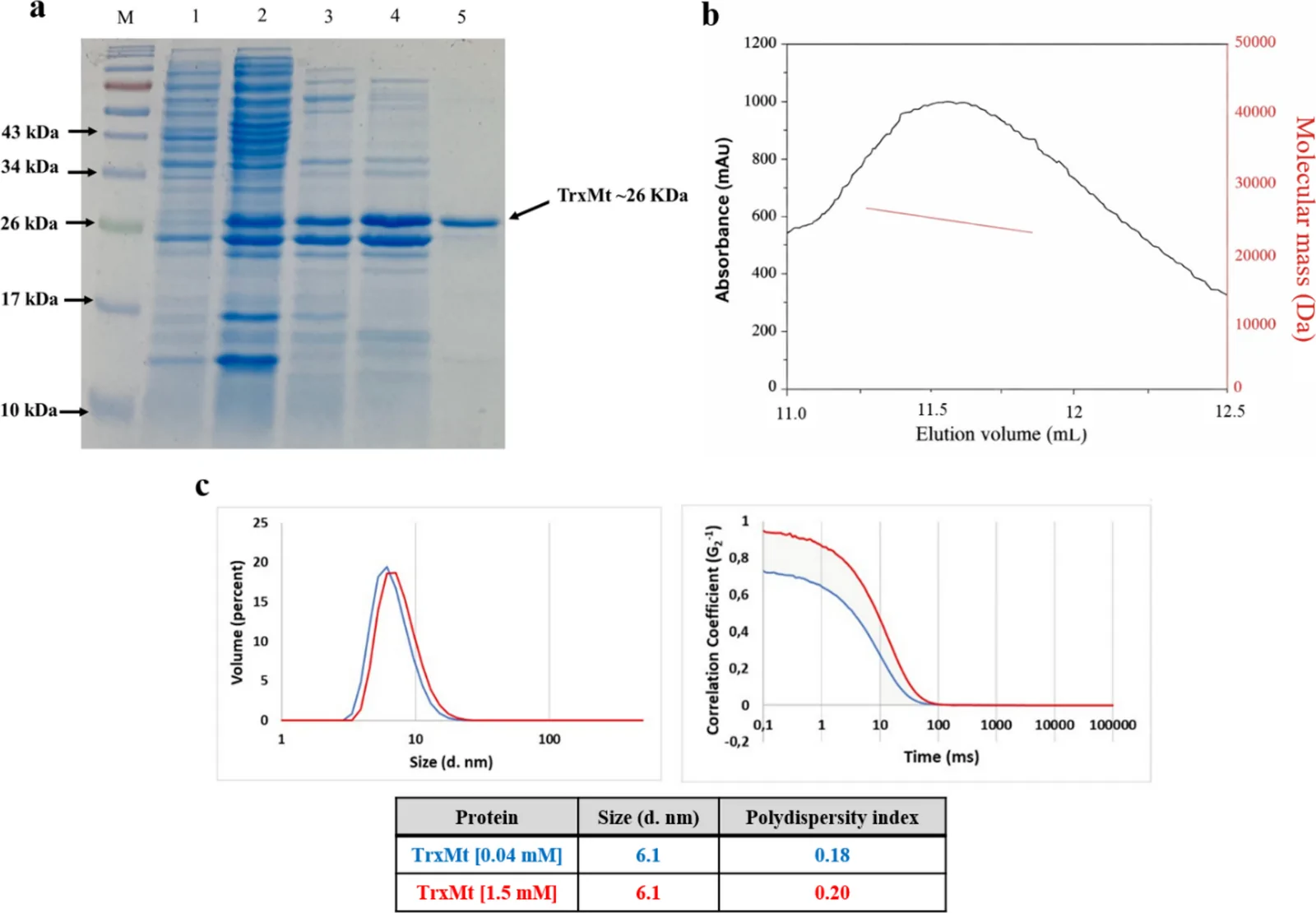
Purification and LS characterization of TrxMt. **a** SDS-PAGE analysis. M, protein marker; lane 1, BL21(DE3) RIL cellular extracts; lane 2, BL21(DE3) RIL cellular extracts after 0.5 mM IPTG induction for 16 h; lane 3, cellular extracts heat-treated at 60 °C; lane 4, fraction purified by anion-exchange; lane 5, fraction purified by IMAC; **b** analysis of purified TrxMt by gel filtration chromatography coupled with SLS. The elution profile of TrxMt is shown as a black line. In red the fitting of TrxMt light scattering points converted to molecular mass; **c** DLS measurements of TrxMt

The protein was purified, almost to homogeneity, by heat treatment, anion-exchange and immobilized metal affinity chromatography (IMAC). As shown in the representative SDS-PAGE analysis (Fig. 3a), TrxMt migrated as a single band with an apparent molecular mass of approximately 26 kDa, consistent with the theoretical molecular weight predicted by ProtParam. The yield of the purified protein was about 4 mg/L of bacterial culture.

The molecular weight of native TrxMt was analyzed through size-exclusion chromatography coupled to multi-angle light scattering (SEC-MALS). This analysis revealed that the protein is monomeric in solution, with an experimental molecular mass of 25,160 ± 150 Da (Fig. 3b). This value is fully consistent with the theoretical molecular weight reported above. Further DLS revealed a hydrodynamic diameter of 6.1 nm, consistent with a small, globular monomeric protein with no significant aggregation under the experimental conditions (Fig. 3c).

### TrxMt disulfide bond reductase activity

**Fig. 4:**
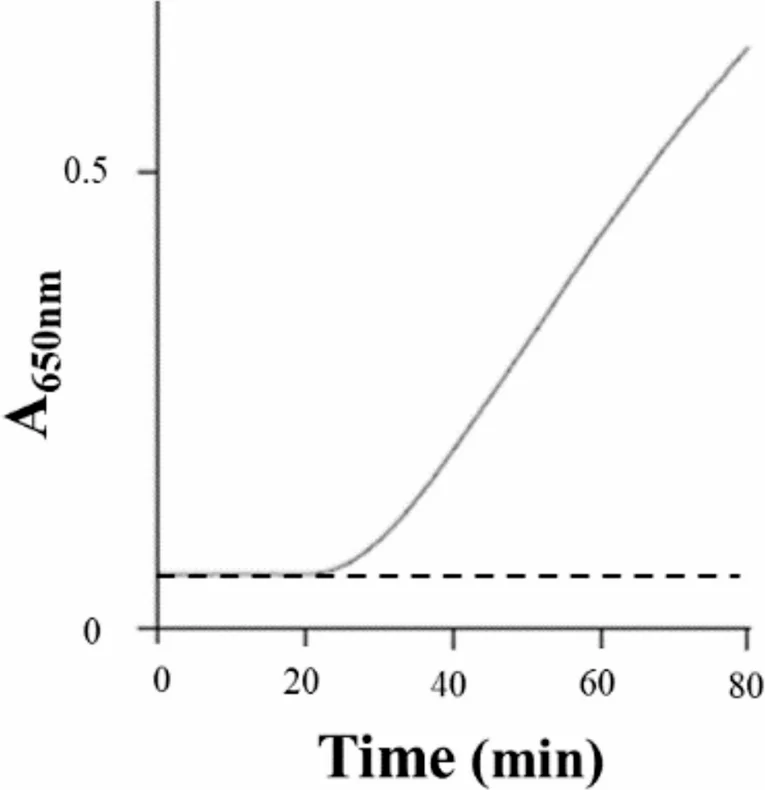
Reduction of bovine insulin disulfides by TrxMt. The reduction assay was performed in the presence of 8 µM apo-TrxMt (black line); the negative control is shown as the dotted line

Since bioinformatic analysis indicated that the N-terminal region of TrxMt adopts a conserved Trx fold containing the canonical C-X-X-C catalytic motif, an insulin disulfide reduction assay was performed to demonstrate Trx activity. TrxMt exhibited disulfide reductase activity, as evidenced by the time-dependent increase in absorbance at 650 nm resulting from precipitation of the insulin B chain (Fig. 4).

### Metal-binding characterization of TrxMt

**Fig. 5:**
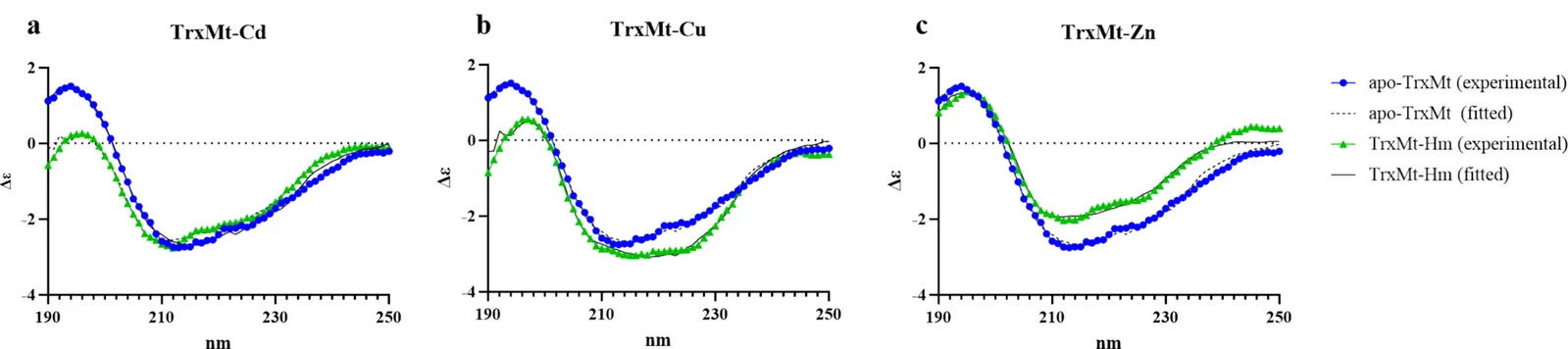
Far-UV CD spectra of TrxMt in presence and in absence of different metal ions. Spectra of TrxMt were recorded with 60 µM Cd^2+^ (**a**), 120 µM Cu^2+^ (**b**), and 80 µM Zn^2+^ (**c**) (green lines) and without metal ions (blue lines)

The metal-binding properties of TrxMt were investigated using far-UV CD and ITC to assess in vitro interaction with Cd^2^⁺, Cu^2^⁺, and Zn^2^⁺. Far-UV CD spectra (190–240 nm) of apo-TrxMt displayed a negative band at 208 nm and a positive band at 194 nm, resulting in a characteristic V-shaped spectrum indicative of a predominantly β-sheet secondary structure (Kuril et al. 2025). Based on preliminary metal titration experiments performed over the concentration range of 40–120 µM, which revealed changes in both the intensity and shape of the CD spectra, metal ion concentrations of 60 µM for Cd^2^⁺, 120 µM for Cu^2^⁺, and 80 µM for Zn^2^⁺ were selected. These concentrations corresponded to the conditions at which no further changes in the protein secondary structure were detected. In the presence of Cd^2^⁺ and Cu^2^⁺ the $\Delta\upvarepsilon$ in the 190- to 200-nm region is reduced compared with the apo-form, suggesting a reorganization of secondary structure while binding of Zn^2^**⁺** leads to more pronounced spectral alterations in the 208- to 222-nm region. This distinct structural response suggests that Zn^2^⁺ interacts with TrxMt through a binding mode different from that of Cd^2^⁺ and Cu^2^⁺, leading to alternative conformational rearrangements of the protein. In contrast, Cu-TrxMt showed a decreasing $\Delta\upvarepsilon$ in the 210- to 230-nm region and a shift in the minima, consistent with larger structural perturbations (Fig. 5).

Quantitative secondary structure analysis was performed using BeStSel algorithms, which are specifically optimized for resolving β-sheet-rich and mixed secondary structure conformations. The analysis confirmed that apo-TrxMt is characterized by a low α-helical content (6.7%) and a substantial β-sheet contribution (29.8%), and a high fraction of unordered structures (49.5%). In the presence of Cd^2^⁺ and Cu^2^⁺ ions, a significant increase in α-helical content was observed with reliable fits, as indicated by low NRMSD values (< 0.1). In contrast, the Zn-TrxMt spectrum resulted in a significant increase in β-sheet and turn content, suggesting a distinct mode of interaction for Zn^2^⁺ compared to the other metal ions (Table 2).

**Table 2:** Secondary structure composition of TrxMt estimated with BeStSel from far-UV CD spectra

	α-helix (%)	β-sheet (%)	Turns (%)	Unordered/others (%)	NRMSD
apo-TrxMt	6.7	29.8	14	49.5	0.02
Cd-TrxMt	20.8	24.6	14.2	40.4	0.03
Cu-TrxMt	15.1	23.1	14.8	46.9	0.03
Zn-TrxMt	5.8	32.3	20.3	41.6	0.04

**Fig. 6:**
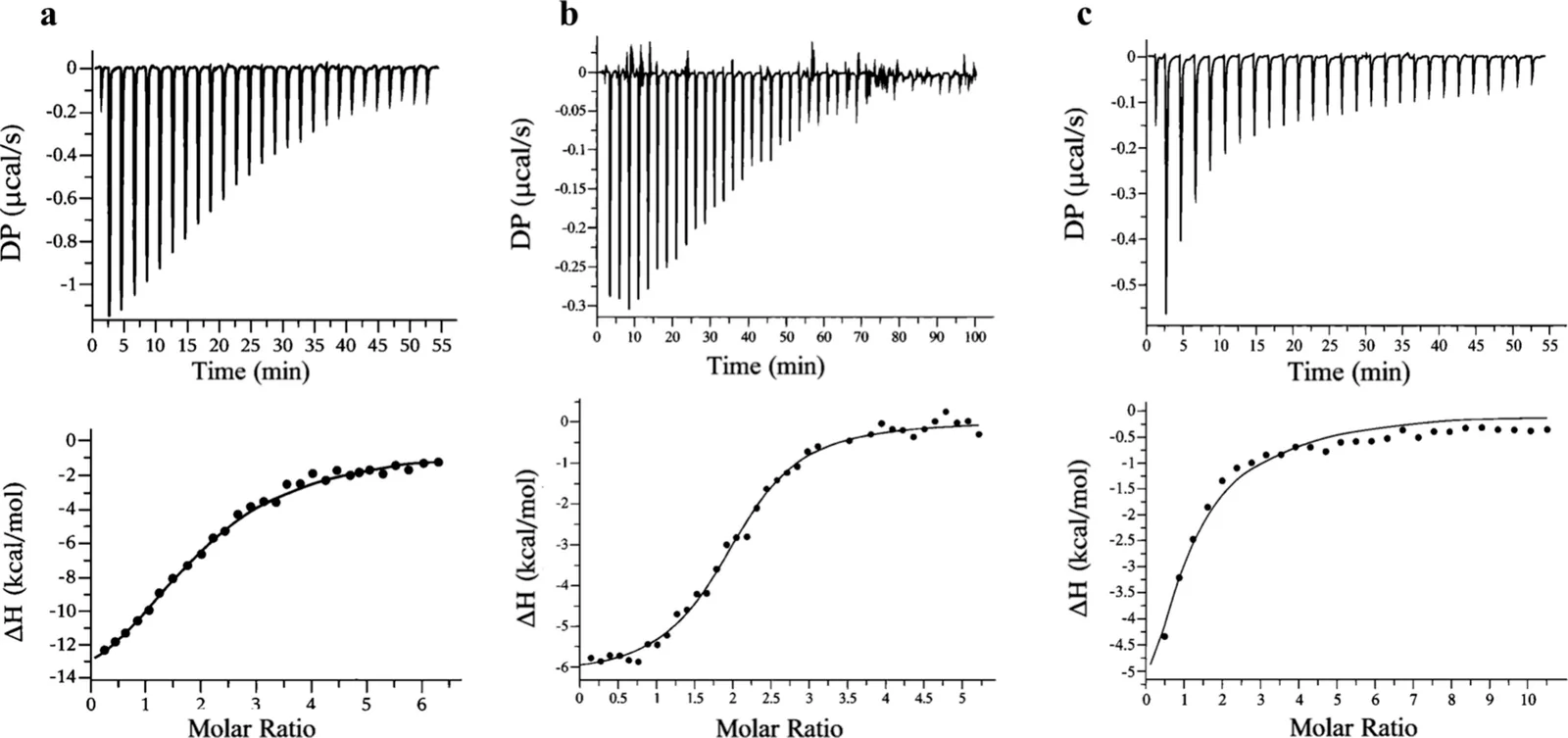
ITC binding studies of TrxMt in the presence of different metal ions. Titration of TrtMt with Cd^2+^ (**a**), Cu^2+^ (**b**), and Zn^2+^ (**c**)

ITC studies were performed to confirm the heavy metal–binding capabilities of TrxMt (Fig. 6). In all cases, TrxMt showed good affinity for the tested metal ions, in the micromolar range, with a preferential binding toward Cu^2+^, as indicated by K_d_ values (Table 3).

**Table 3:** Thermodynamic parameters derived from ITC binding experiments between TrxMt and metal ions

Model: One set of sites					
Ligand	*K*_d_ (µM)	Δ*G* (kcal/mol)	ΔH (kcal/mol)	−TΔS(kcal/mol)	*n*
CdCl_2_	19.6 ± 1.7	−6.4	−19.8 ± 1.0	13.4	1.1 ± 0.3
CuCl_2_	2.2 ± 0.2	−7.7	−6.2 ± 0.1	−1.5	1.1 ± 0.1
ZnCl_2_	27.3 ± 5.3	−6.2	−12.5 ± 4.1	6.3	0.9 ± 0.3

*K*_d_ and ΔH values were determined from MicroCal PEAQ ITC analyses software fitting data using the model one set of site for Cd^2+^, Cu^2+^, and Zn^2+^ metal ions

#### TrxMt enhance tolerance in *E. coli* against HMs toxicity

**Fig. 7:**
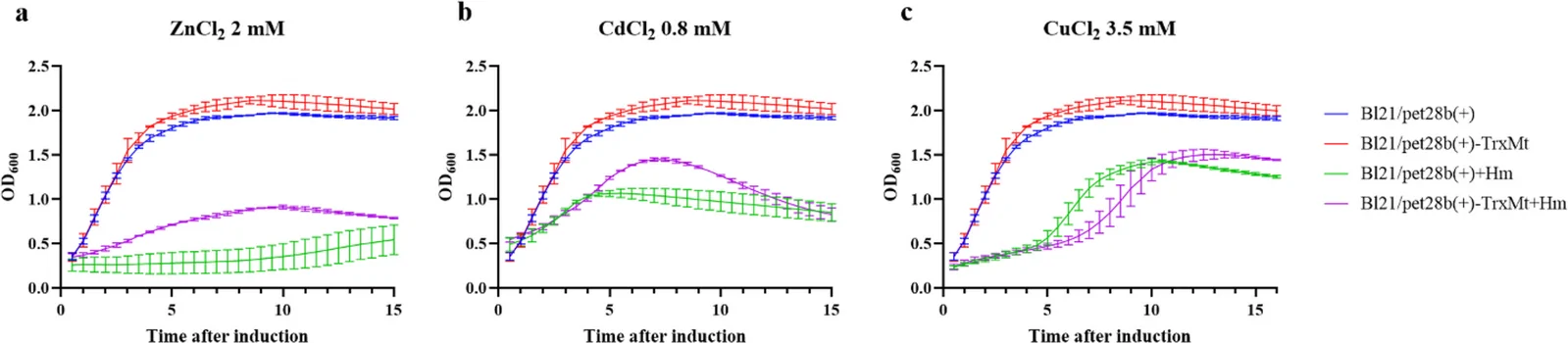
Growth curves of *E. coli* cells expressing TrxMt in the presence of 2 mM ZnCl_2_ (**a**), 0.8 mM CdCl_2_ (**b**) and 3.5 mM CuCl_2_ (**c**). BL21(DE3)RIL/pET28b(+)-TrxMt and BL21(DE3)RIL/pET28b(+) exposed to Hm are shown in purple and green, respectively, while the controls BL21(DE3)RIL/pET28b(+)-TrxMt and BL21(DE3)RIL/pET28b(+) are shown in red and blue, respectively. Data are presented as mean ± SD of three independent biological replicates

Preliminary MIC assays using metal ion concentrations ranging from 0.5 to 5 mM were performed to determine the concentrations that inhibit bacterial growth. The MIC values for BL21(DE3)RIL/pET28b(+) were 2 mM for CdCl_2_, 5 mM for CuCl_2_, and 2.5 mM for ZnCl_2_, whereas the corresponding MIC values for BL21(DE3)RIL/pET28b(+)-TrxMt were 2 mM, 5 mM, and 4 mM, respectively. Based on these results, growth kinetics assays were performed at subinhibitory concentrations of 0.8 mM CdCl_2_, 3.5 mM CuCl_2_, and 2 mM ZnCl_2_ to evaluate whether TrxMt expression enhances metal tolerance in BL21(DE3)RIL/pET28b(+)-TrxMt compared with the control strain, BL21(DE3)RIL/pET28b(+). Among the tested conditions, exposure to 2 mM ZnCl_2_ resulted in improved growth during the induction phase in the TrxMt-expressing strain (Fig. 7a). In contrast, under exposure to 0.8 mM CdCl_2_, the TrxMt-expressing strain displayed enhanced tolerance compared with the control strain only during the protein induction period (5–10 h). (Fig. 7b); under 3.5 mM CuCl_2_ treatment, only a slight difference between the TrxMt-expressing and control strains was observed after 16 h (Fig. 7c).

## Discussion

In this study, we identified TrxA, a novel chimeric protein from *R. aurantiaca*, composed of an N-terminal Trx domain fused to a C-terminal Mt domain. This protein was characterized through different approaches, including in silico analyses, expression and purification of the recombinant protein (named TrxMt), Trx activity assays, and evaluation of the protein’s ability to bind different metal ions. In silico analyses based on BLASTp and ClustalW alignments indicated homology between the N-terminal domain and bacterial Trx proteins, as well as between the C-terminal region and bacterial Mt proteins. Both domains display their characteristic signatures: the presence of the canonical CXXC catalytic motif in the Trx fold and the hallmark features of bacterial Mts, including a high cysteine content and conserved residues. A synthetic gene optimized for E. coli codon usage was expressed, and the recombinant protein TrxMt was purified and biochemically characterized. The protein was stable, in agreement with the bioinformatic predictions; indeed, the II of Mt domain of TrxA is 31.49 while the II of the whole protein is 20.98 suggesting that the presence of Trx domain may stabilize the overall protein structure. The Trx fold is widely used as a fusion partner in recombinant protein expression because its compact, hydrophilic, and thermostable structure promotes correct folding and reduces aggregation (Pedone et al. 2010). In contrast, Mts are generally less stable and often require fusion tags, such as GST (Shahpiri and Mohammadzadeh 2018) or SUMO (Li et al. 2021), to improve solubility and stability. Indeed, the untagged Mts from an *M. cyanobacterium* (MGF1494285) and *S. elongatus* (WP_011242577) exhibit relatively high II of 32.23 and 38.83, respectively, according to ProtParam predictions. Furthermore, the presence of a Trx domain in hybrid redox-sensitive proteins may facilitate the reversible oxidation of cysteine residues. Chimeric proteins containing a Trx fold fused to other domains have previously been reported. For example, in *Haemophilus influenzae*, *Vibrio cholerae*, and *Neisseria meningitidis*, hybrid proteins have been described in which an antioxidant peroxiredoxin domain is linked to a Trx fold responsible for its regeneration (Pedone et al. 2010). Similarly, in TrxA, the Trx domain may stabilize the hybrid protein and maintain the cysteine residues of the Mt domain in a reduced state. Furthermore, DLS analysis (data not shown) confirmed the remarkable solubility of TrxMt, with the protein remaining soluble even at concentrations as high as [1.5 mM]. Altogether, these observations suggest that TrxA is a naturally occurring chimeric protein in which a Trx domain is fused to an Mt domain, providing enhanced structural stability and potential functional advantages.

Functional assays confirmed that the recombinant protein TrxMt exhibits disulfide reductase activity comparable to that reported for other Trxs (Holmgren 1979), indicating that the Trx domain remains catalytically active within the chimeric protein.

To evaluate the metal-binding ability of TrxMt, CD and ITC analyses were performed. CD spectroscopy revealed that TrxMt undergoes conformational changes upon metal binding, suggesting the occurrence of metal coordination. This interaction was further confirmed by ITC experiments, which indicated the binding of Cd^2^⁺, Cu^2^⁺, and Zn^2^⁺ with micromolar affinity, consistent with a single-site binding model. However, the observed K_d_ values were higher than those generally reported for previously characterized eukaryotic and prokaryotic Mts (Mosna et al. 2023), which typically exhibit nanomolar to picomolar metal-binding affinities, indicative of substantially weaker metal–protein interactions. Further studies based on site-directed mutagenesis will be necessary to identify the residues involved in metal coordination and to clarify the specific contribution of the Mt domain to metal binding.

Finally, the functional role of TrxMt in metal tolerance was further investigated through in vivo experiments. Expression of TrxMt in *E. coli* increased tolerance to Zn^2^⁺ compared with control cells. In contrast, in the presence of 0.8 mM CdCl_2_ and 3.5 mM CuCl_2_, the TrxMt-expressing strain exhibited only a slight increase in tolerance compared with the control strain, observed during the protein induction period (5–10 h) for Cd^2^⁺ and after 16 h of incubation for Cu^2^⁺. Further studies are needed to elucidate the molecular mechanisms underlying heavy metal tolerance and homeostasis in recombinant bacteria expressing TrxMt. Regarding bacterial Mts, to our knowledge, limited quantitative data are available for direct comparison. For example, in *Synechococcus* PCC 7942 (SmtA) (Seifipour et al. 2017), bioassays showed that recombinant bacteria were able to grow up to 0.7 mM CdCl_2_ in the presence of IPTG within 16 h, whereas previous studies on NmtA from* Anabaena *PCC 7120 reported lower cadmium tolerance (0.5 mM) (T. V. et al. 2018). These differences could likely reflect variations in protein architecture, metal-binding properties, and cellular expression context, rather than directly comparable intrinsic affinities.

## Conclusion

A novel chimeric protein, TrxA, identified from R. aurantiaca, was successfully produced as a recombinant protein (TrxMt) in *E. coli*. TrxMt represents an unusual example of a naturally occurring fusion between two functional modules typically found as independent proteins. Biochemical and biophysical characterization indicated that TrxMt is a soluble monomer, structurally stable, endowed with disulfide reductase activity and capable of binding different HMs. Based on our results, the affinity toward the different HMs analyzed may be influenced by the Mt copy number, as previously reported for tandem Mt constructs (Ma et al. 2019).

Overall, these findings provide a preliminary framework for future investigations. Further research should aim to elucidate the molecular basis of TrxMt function by exploring the relationship between its metal-binding properties and redox activity, two processes that are linked during heavy metal stress response. Additional biochemical and structural studies will be necessary to clarify how the Trx and Mt domains contribute to the overall properties of the protein and to better define the functional significance of their association within a single polypeptide. This knowledge could also enhance the applicability of TrxMt-based systems for metal biosensing, detoxification, and bioremediation applications.

## Supplementary information

Below is the link to the electronic supplementary material.

ESM 1 (PDF 178 KB)

## Data Availability

No datasets were generated or analyzed during the current study.
