# Supplementary material for: Identification and characterization of TrxA: a novel bacterial thioredoxin–metallothionein chimera
Source: Appl Microbiol Biotechnol. 2026 Aug 29;110:273. doi: 10.1007/s00253-026-14002-w (PMC13578184; doi:10.1007/s00253-026-14002-w)
Supplement: Supplementary file 1 [file 253_2026_14002_MOESM1_ESM.pdf]

Identification and characterization of TrxA: a novel bacterial thioredoxin–metallothionein chimera

Annamaria Vitiello[1], Luciano Pirone[2], Martina Filocaso[2,3], Gabriella Fiorentino[1], Emilia Pedone2*, Danila Limauro1*

[1]Department of Biology, University of Naples Federico II, Via Cinthia, 89126, Naples, Italy; fiogabri@unina.it; annamaria.vitiello2@unina.it
[2]Institute Biostructures and Bioimaging, C.N.R., Via Pietro Castellino 111, 80131 Naples, Italy; luciano.pirone@cnr.it;
[3]Department of Environmental, Biological and Pharmaceutical Sciences and Technologies, University of Campania "Luigi Vanvitelli", 81100 Caserta, Italy;
martina.filocaso@unicampania.it

*Correspondence: limauro@unina.it; emilia.pedone@cnr.it

Supplementary Information n.1
Codon-optimized nucleotide sequence of TrxMt
ATGGGCAGCAGCCATCATCATCATCATCACAGCAGCGGCCTGGTGCCGCGCGGCAGCCATATGTCAGA
TAAAATAATTCACCTAACAGATGACTCGTTCGATACCGATGTGCTGAAAGCGGACGGCGCGATTTTGGT
AGATTTCTGGGCTGAATGGTGTGGTCCGTGTAAAATGATTGCGCCGATTCTGGACGAGATCGCCGACG
AGTATCAGGGTAAATTGACCGTTGCAAAATTGAATATCGACCAAAACCCAGGTACGGCTCCGAAGTAT
GGTATTCGTGGTATCCCGACCCTGCTTTTATTTAAGAATGGTGAAGTTGCAGCGACCAAAGTGGGTGCT
CTGAGCAAGGGTCAGCTGAAGGAGTTCCTGGATGCAAATCTGGCGGGCTCCGGCTCTGGTCACATGC
ATCACCACCACCACCACAGCTCCGGTCTGGTTCCGCGTGGTAGTGGCATGAAAGAGACTGCCGCCGC
GAAGTTTGAGCGTCAACACATGGATAGCCCGGATCTCGGTACGGATGACGACGACAAGGCTATGGCAG
ACATCGGCTCCATGACCAGCACCACCCTGGTCAAATGCGCCTGCGAACCGTGTCTGTGCAACGTGGAT
CCGAGCAAGGCGATCGACCGCAACGGCCTGTACTACTGCAGCGAAGCGTGCGCGGATGGCCACACGG
GCGGCTCTAAGGGGTGCGGTCATACCGGCTGCAACTGCCATGGTTAACTCGAG-
